# Clinicopathological landscape and management trends of thyroid carcinoma over two decades: a single-institution study and risk stratification of central lymph node metastasis in T1 papillary thyroid carcinoma

**DOI:** 10.3389/fendo.2026.1776293

**Published:** 2026-04-30

**Authors:** Li’nan Qin, Xindi Ke, Yongdu Nie, Yiming Zhu, Shaoyan Liu, Jian Wang

**Affiliations:** Department of Head and Neck Surgical Oncology, National Cancer Center/National Clinical Research Center for Cancer/Cancer Hospital, Chinese Academy of Medical Sciences and Peking Union Medical College, Beijing, China

**Keywords:** central lymph node metastasis, nomogram, Papillary thyroid carcinoma, risk stratification, thyroid cancer trends

## Abstract

**Purpose:**

This study aimed to describe the clinicopathological landscape of thyroid disease, summarize long-term management trends of thyroid carcinoma (TC) and lymph node metastasis (LNM) from 1999 to 2018, and develop a nomogram to predict central lymph node metastasis (CLNM) in patients with T1-stage papillary thyroid carcinoma (PTC).

**Methods:**

A total of 34,420 patients with thyroid disease were included for landscape analysis, among whom 18,917 patients with TC were analyzed for management trends. Subsequently, 11,760 patients with T1-stage PTC were enrolled to identify risk factors for CLNM. A predictive nomogram was constructed using multivariable logistic regression and internally validated.

**Results:**

Among all patients, 22,919 were diagnosed with thyroid cancer and 11,501 with benign nodules. The proportion of TC increased steadily, reaching 99.3% in 2018. PTC accounted for 99.3% of TC cases, while the proportion of papillary thyroid microcarcinoma showed a slight decline. The rate of central lymph node dissection increased from 34.4% to 97%, whereas the CLNM rate decreased from 81.8% to 55%. In contrast, lateral lymph node dissection declined from 58.3% to 28.8%, with a corresponding reduction in lateral lymph node metastasis. Younger age, male sex, larger maximum tumor diameter (MTD), multifocality, lymphovascular invasion, and capsule invasion were identified as independent risk factors for CLNM (all p<0.05). The optimal MTD cutoff was 0.75 cm. The nomogram showed good discrimination (C-index 0.715) and satisfactory calibration.

**Conclusions:**

This study demonstrates evolving patterns and increasing standardization in thyroid cancer management over two decades. The proposed nomogram may serve as a postoperative pathology-based risk stratification tool to support individualized management and follow-up in patients with T1-stage PTC.

## 1 Introduction

In recent years, the incidence of thyroid cancer has risen significantly. Thyroid cancer is now the 7th most common cancer in China and the 3rd most common among Chinese women (1). According to the SEER database, the estimated number of new cases in the United States in 2022 was 43,800, with 2,230 estimated deaths and a 5-year relative survival rate of 98.4%. Papillary thyroid cancer (PTC) accounts for more than 90% of all thyroid cancer and is generally associated with a favorable prognosis. It is reported that lymph node metastasis (LNM) is an indicator of poor survival and high recurrence rate (2–4), with an incidence of approximately 40% (5).

Surgery is the primary treatment strategy. While complete dissection of cervical lymph node may reduce recurrence risk and avoid the need for postoperative radioactive iodine (RAI) therapy (2, 6, 7), prophylactic central lymph node dissection (CLND) remains controversial. Some studies suggest that prophylactic dissection does not significantly increase outcome (7, 8) and carries risks of complications, including hypocalcemia and recurrent laryngeal nerve injury. Consequently, current guideline often recommends against prophylactic CLND for patients with cT1-2N0, noninvasive PTC (2).

Due to the lack of standardized protocols in past decades, TC surgery practices varied dramatically across institutions. Management of the primary tumor and cervical lymph node was inconsistent, potentially leading to poor patient outcomes. In recent years, with the promotion of American Thyroid Association (ATA) guidelines and Chinese expert consensus and guidelines on TC treatment, surgical practices have gradually become standardized. Specifically, the proportion of CLND has gradually increased, while the proportion of lateral neck lymph node dissection (LLND) has decreased (5).

With the increasing detection of early-stage papillary thyroid carcinoma, accurate assessment of lymph node status has become an important clinical issue. Lymph node metastasis is associated with pathological upstaging and may influence postoperative management and follow-up strategies in patients with differentiated thyroid cancer. In clinical practice, ultrasonography and computed tomography are routinely used for cervical lymph node evaluation. However, microscopic lymph node metastases, particularly in the central compartment, may remain undetectable with preoperative imaging. Consequently, occult central lymph node metastasis continues to be a frequent pathological finding, even in patients with small, early-stage tumors. Therefore, improved characterization of clinicopathological factors associated with occult central lymph node metastasis remains clinically relevant, particularly for postoperative risk stratification and individualized follow-up planning in patients with early-stage papillary thyroid carcinoma.

In this study, we retrospectively analyzed a large cohort of patients treated at a national cancer center over a 20-year period to describe the evolving clinicopathological landscape and management trends of thyroid carcinoma. In addition, we developed and internally validated a nomogram to estimate the risk of central lymph node metastasis in patients with T1-stage papillary thyroid carcinoma, with the aim of complementing conventional pathological staging and assisting postoperative risk assessment.

## 2 Materials and methods

### 2.1 Study population

**Figure 1:**
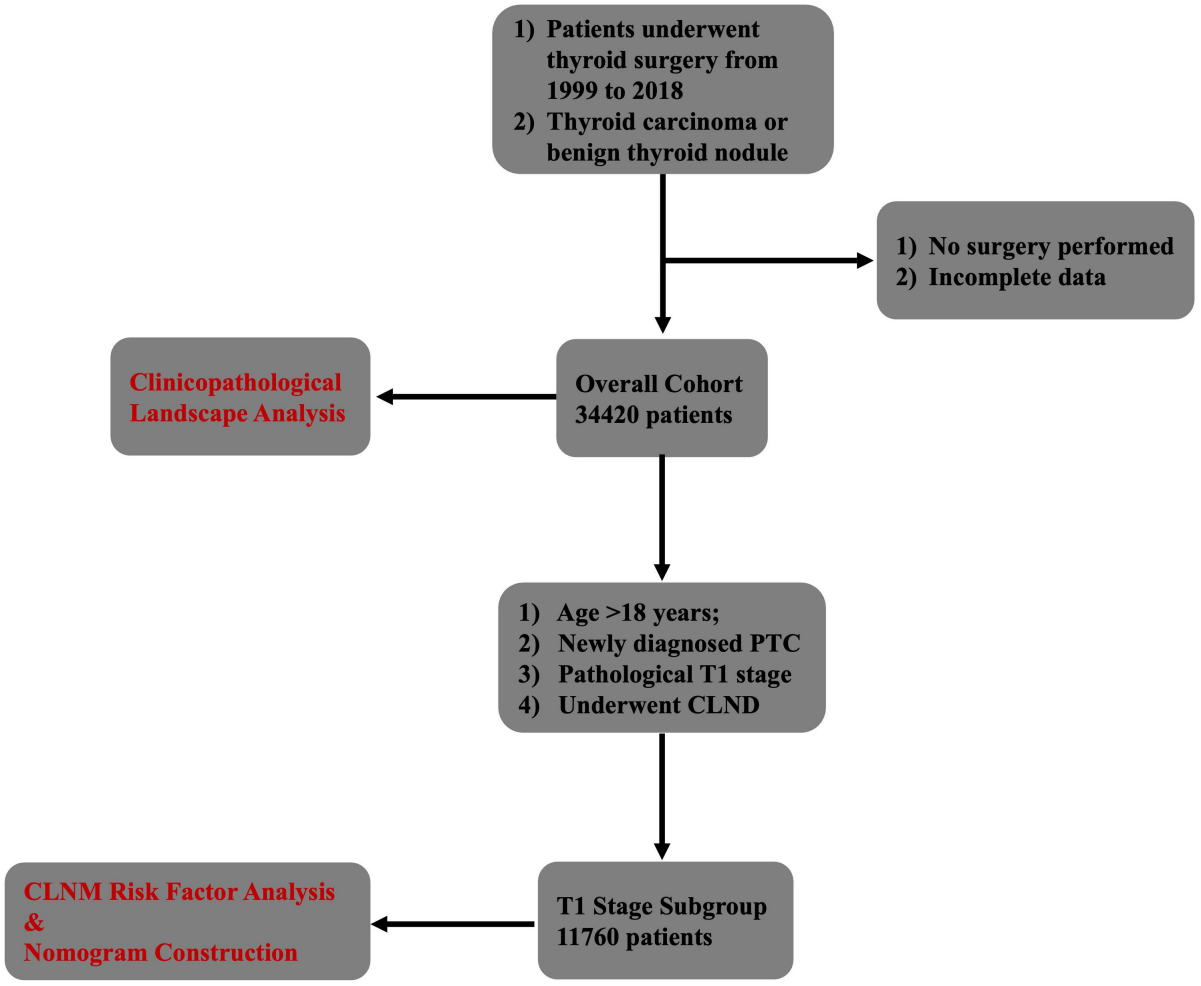
Flowchart of the inclusion and exclusion criteria.

We retrospectively reviewed patients who underwent surgery for thyroid disease at Cancer Hospital, Chinese Academy of Medical Sciences (CAMS) from 1999 to 2018. As illustrated in the study flowchart (**Figure 1**), this study was designed with two main perspectives, utilizing two distinct cohorts.

For the clinicopathological landscape and management trend analyses, the inclusion criteria were: (1) patients who underwent thyroid surgery in our institution during the study period; and (2) pathological diagnosis of either thyroid carcinoma or benign thyroid nodule. The exclusion criteria were: (1) no surgical treatment performed at our institution; and (2) incomplete clinical data.

For the analysis of risk factors and nomogram construction for central lymph node metastasis (CLNM), a separate subgroup was defined. The inclusion criteria for this subgroup were: (1) age >18 years; (2) newly diagnosed papillary thyroid carcinoma (PTC); (3) pathological T1 stage according to the 8th AJCC TNM staging system; and (4) underwent central lymph node dissection (CLND).

This study was approved by the institutional Ethics Committee of Cancer Hospital, CAMS.

### 2.2 Clinicopathological variables

Clinical variables, including age and gender, were retrieved from medical records. The pathological data including maximum tumor diameter (MTD), histological type, LNM status, and the presence of multifocality (MF), capsule invasion (CI), Hashimoto’s thyroiditis (HT), and lymph-vascular invasion (LVI) were retrieved from the pathological report. MF was defined as more than one tumor focus within the thyroid. CI was defined as microscopic tumor invasion into the thyroid capsule. HT was defined by diffuse infiltration of lymphocytes, plasma cells, and oxyphilic cells, with lymphoid follicles and reactive germinal centers in a normal region of the thyroid gland distinct from cancer foci (9). LVI was defined as the presence of tumor cells within lymph ducts or blood vessels under the microscope.

### 2.3 Quality control

The surgeries were performed by senior surgeons in the Department of Head and Neck Surgical Oncology at Cancer Hospital, CAMS. The surgical plans were discussed by at least two senior surgeons prior to operation. The pathological reports were reviewed by two pathologists in the Department of Pathology at Cancer Hospital, CAMS. The data collection and analysis were conducted independently by two researchers.

### 2.4 Statical analysis

The mean and standard deviation (SD) values were calculated for continuous variable (age, MTD). The Chi-square test was used to compare categorical variables. Univariable logistic regression was utilized to screen for candidate risk factors, followed by multivariable logistic regression to identify independent predictors and construct the CLNM prediction model. The continuous variable MTD was converted into a categorical variable, and the optimal cutoff value was determined by the maximum Youden’s index. The descriptive figures were generated using Prism ver. 9.0.0 (GraphPad Software, LLC). All statistical analysis was performed using R ver. 3.6.3 (R-project, Institute for Statistics and Mathematics, Vienna, Austria).

## 3 Results

### 3.1 Basic information

From 1999 to 2018, a total of 34,420 patients underwent surgery for thyroid disease in our institution. This overall cohort was used for clinicopathological landscape analysis, while a separate subgroup of 11,760 adult patients with newly diagnosed T1-stage PTC who underwent CLND was used for CLNM risk factor analysis and nomogram construction. Of these, 11,501 patients were diagnosed with benign thyroid nodules, and 22,919 patients were pathologically diagnosed as TC. Among all patients with TC, 18,917 patients were treated in our institution for the first time. Regarding pathological type, PTC accounted for 99.3%(n=18785/18917), medullary thyroid cancer (MTC) for 0.2%(n=37/18917), and follicular thyroid cancer (FTC) for 0.1% (n=19/18917) (**Table 1**). Among patients with benign nodules, the male-to-female ratio was approximately 1:1, whereas for PTC patients (**Table 2**), it was approximately 1:2.8, indicating a significant difference. In terms of age distribution, the average age of patients with TC was 43.9 years. For males, ages ranged from 6 to 87 years (median: 43); for females, ages ranged from 6 to 84 years (median:44), without significant difference in age distribution. Detailed yearly data is provided in **Supplementary Table 1**.

**Table 1:** Basic information of the overall cohort.

Characteristics of overall cohort	N=34420
TC	
Yes	22919 (66.6%)
No	11501 (33.4%)
First treated TC	
Yes	18917 (82.5%)
No	4002 (17.5%)
Histological type in first treated TC	
PTC	18785 (99.3%)
MTC	37 (0.2%)
FTC	18 (0.1%)
PDC	24 (0.1%)
ATC	28 (0.1%)
PTC+MTC	2 (0.01%)
PTC+FTC	23 (0.1%)

PTC, FTC, MTC, PDC, and ATC account for papillary thyroid carcinoma, follicular thyroid carcinoma, medullary thyroid carcinoma, poorly differentiated thyroid carcinoma, and anaplastic thyroid carcinoma, respectively. The percentage of histological type was calculated among all the 18,917 patients treated in our institution for the first time.

**Table 2:** Clinicopathological features of first treated PTC patients.

Clinicopathological features of first treated PTC patients	
Gender	
Male	4908 (26.1%)
Female	13877 (73.9%)
Age/yo	
Mean	43.9
SD	11.1
MTD/cm	
Mean	1.1
SD	1.2
PTMC	
Yes	16510 (87.9%)
No	2275 (12.1%)
Lymph node	
CLNM	8710 (46.4%)
CLND	15661 (83.4%)
LLNM	3925 (20.9%)
LLND	5666 (30.2%)
MF	
Yes	16001 (85.2%)
No	2784 (14.6%)
CI	
Yes	10172 (54.1%)
No	8613 (45.9%)
LVI	
Yes	244 (1.3%)
No	18541 (98.7%)
HT	
Yes	5091 (27.1%)
No	13694 (72.9%)

SD accounts for standard deviation. MTD accounts for maximum tumor diameter. PTMC accounts for papillary thyroid microcarcinoma. CLNM, CLND, LLNM, and LLND account for central compartment lymph node metastasis, central compartment lymph node dissection, lateral compartment lymph node metastasis, and lateral compartment lymph node dissection, respectively. MF, CI, LVI, HT account for multifocality, capsule invasion, lymph-vascular invasion, and Hashimoto’s thyroiditis, respectively.

Pathological PTC subtype information was provided to 571 patients from 1999 to 2015, and 1,411, 1,921, and 3566 patients from 2016, 2017, and 2018, respectively. We analyzed the distribution of pathological PTC subtypes in these 7,469 patients. The conventional subtype was most prevalent (67.06%), followed by the follicular variant (30.43%). The other subtypes (oncocytic, solid, tall cell, diffuse sclerosing, clear cell, and columnar cell) each accounted for less than 1% (**Supplementary Figure 1**).

### 3.2 Trends in thyroid surgery volume and TC proportion

**Figure 2:**
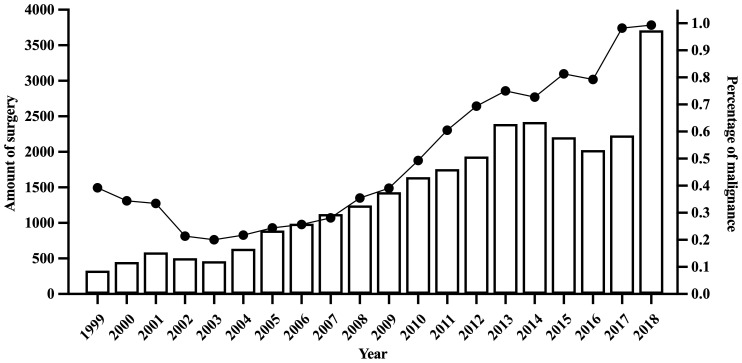
Trends of surgery volume and thyroid cancer proportion from 1999 to 2018.

**Figure 2** showed the trends in thyroid surgery volume and TC proportion from 1999 to 2018. The annual number of thyroid surgeries increased steadily from 329 in 1999 to 3,774 in 2018. The proportion of TC decreased from 39.2% (n=129/329) in 1999 to 20.0% (n=97/484) in 2003, then rose rapidly to 99.3%(n=3748/3774) in 2018.

### 3.3 Trends of PTC and PTMC proportion

**Figure 3:**
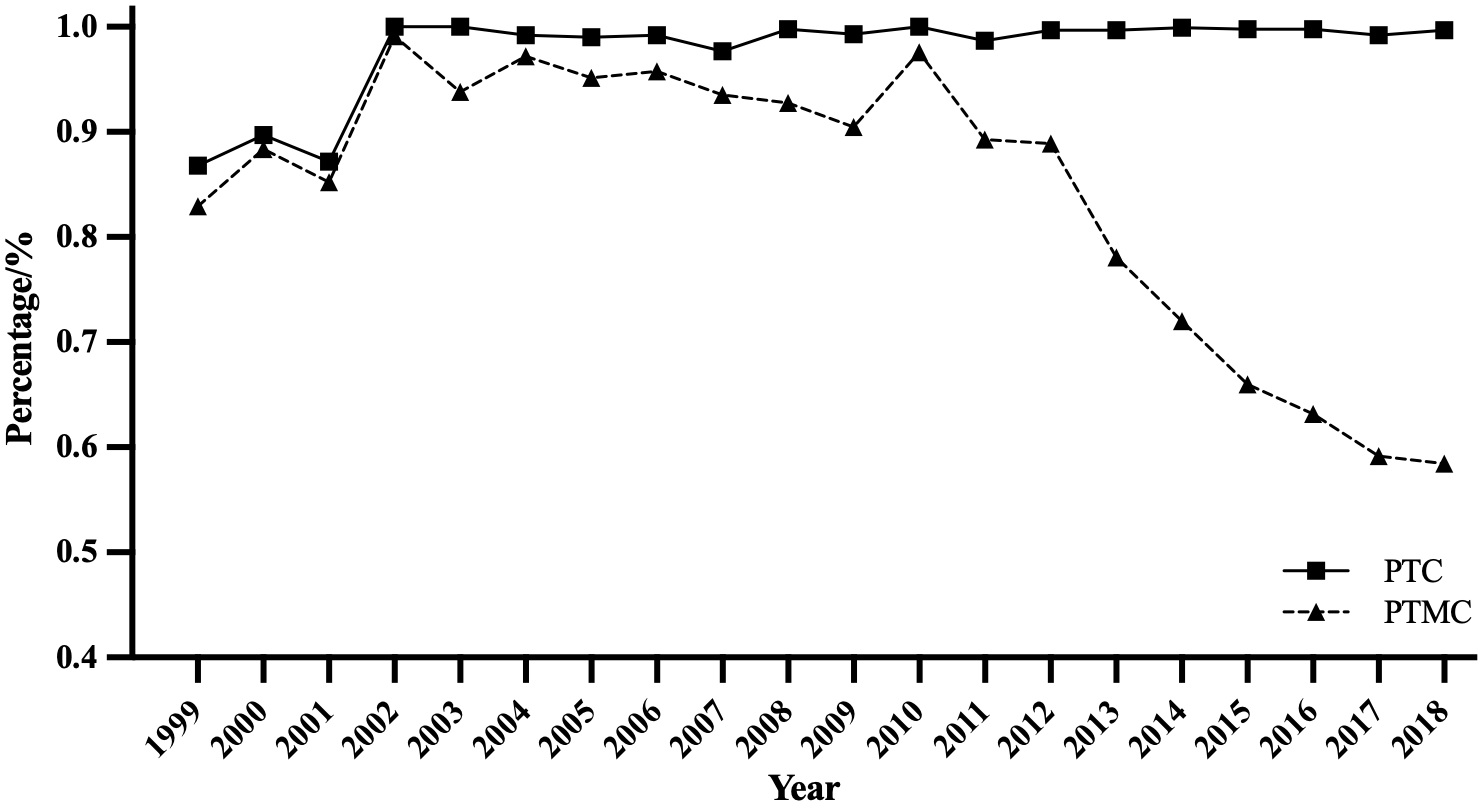
Trend of PTC and PTMC proportion from 1999 to 2018. (PTC and PTMC account for papillary thyroid carcinoma and papillary thyroid microcarcinoma).

As shown in **Figure 3**, among all newly diagnosed TC patients, the proportion of PTC has been increasing rapidly and remained stable at over 99%. The proportion of PTMC fluctuated around 90% from 1999 to 2010, then rapidly declined to 58% between 2010 and 2018.

### 3.4 Trends of LND and LNM proportion

**Figure 4:**
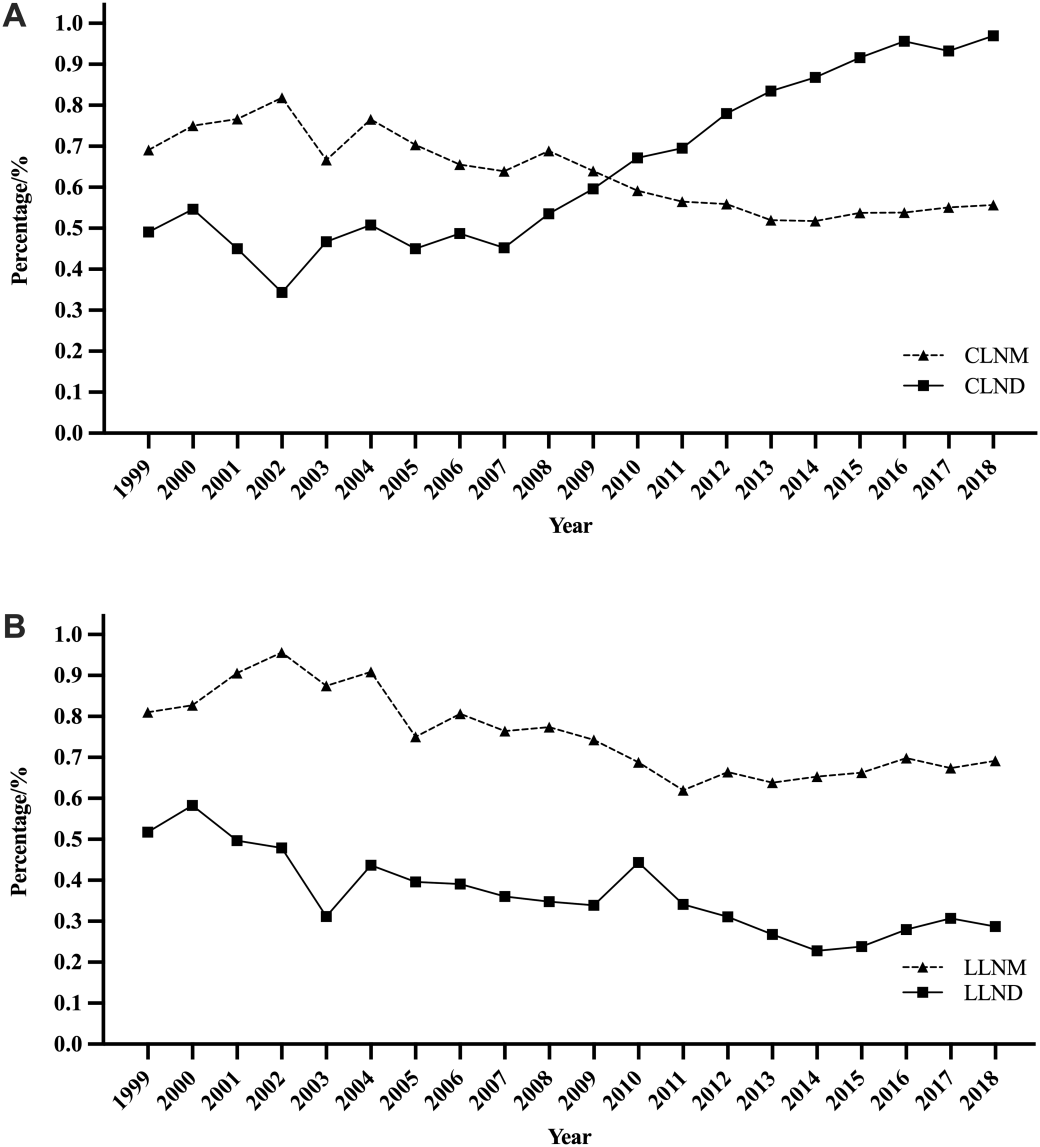
Proportion trend of lymph node dissection and lymph node metastasis. Panel **(A)** for central compartment lymph node dissection and metastasis. Panel **(B)** for lateral compartment lymph node dissection and metastasis. (CLNM, CLND, LLNM, and LLND account for central compartment lymph node metastasis, central compartment lymph node dissection, lateral compartment lymph node metastasis, and lateral compartment lymph node dissection, respectively).

The proportion trends of LND and LNM are shown in **Figure 4**. The proportion of CLND gradually increased from the lowest level of 34.4% (n=33/96) in 2002 to 96.7% (n=3568/3690) in 2018 (**Figure 4A**). During the same period, the proportion of pathologically confirmed CLNM among patients who underwent CLND decreased from 81.8% (n=27/33) in 2002 to 55.7% (n=1988/3568) in 2018. This finding should be interpreted in the context of the expanding use of prophylactic CLND over time. The rate of LLND (including level II, III, IV, and V) showed an overall downward trend, from 52.3% (n=81/155) in 2000 to 28.7% (n=1058/3690) in 2018 (**Figure 4B**). Similarly, the proportion of LLNM gradually decreased from the highest of 95.7% (n=44/46) in 2002 to 69.2% (n=732/1058) in 2018.

### 3.5 Risk factors analysis of CLNM in patients with T1-stage PTC

**Figure 5:**
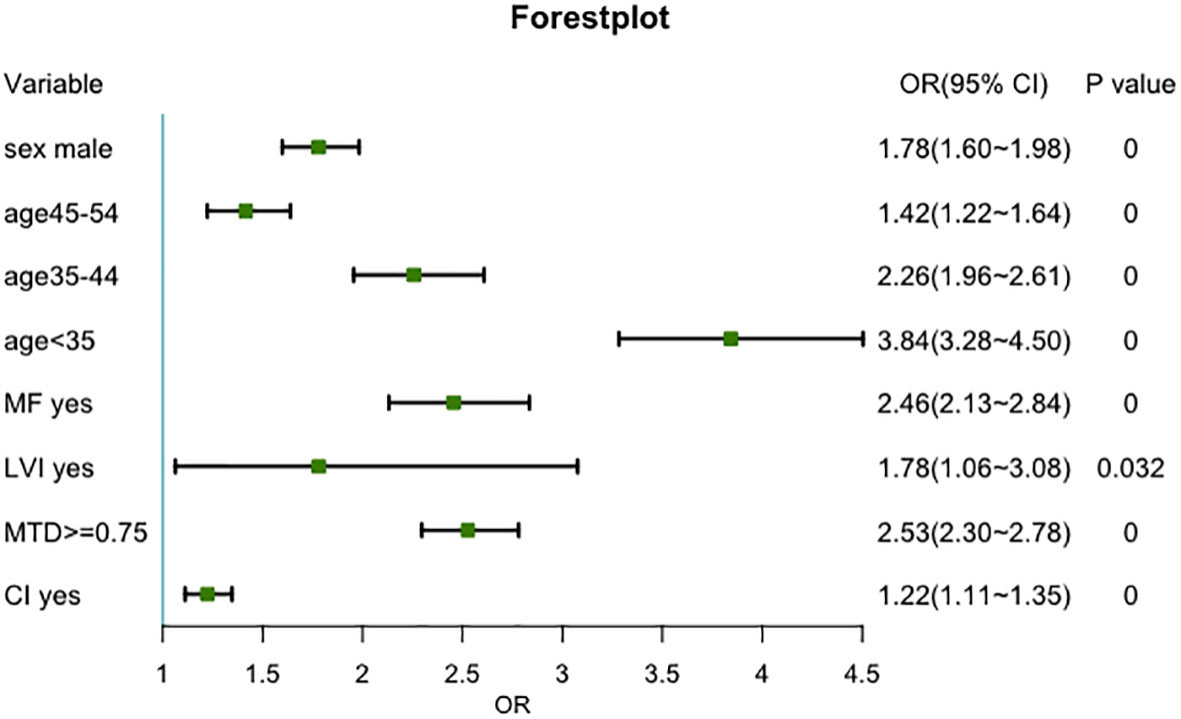
Forest plot of the risk factors analysis for central compartment lymph node metastasis. (MF, LVI, MTD and CI account for multifocality, lymph-vascular invasion, maximum tumor diameter, and capsule invasion, respectively.).

A total of 11,760 patients were included according to the inclusion criteria mentioned above. The cutoff value of MTD was determined according to the maximum Youden’s index. The ROC curve between CLNM and MTD was shown in **Supplementary Figure 2**, and the optimal cutoff value of MTD was 0.75cm (Sensitivity=0.592, Specificity=0.648, AUC = 0.659, p<0.05). The variables distribution was shown in **Table 3**. The univariable analysis showed that younger age, male, MTD ≥ 0.75 cm, and the presence of MF, LVI, and CI were significantly associated with an elevated risk of CLNM, while HT was not significantly associated with CLNM (p=0.245). The multivariable analysis confirmed that younger age, male, MTD ≥ 0.75 cm, MF, LVI, and CI were independent risk factors for CLNM (**Figure 5**).

**Table 3:** Variables distribution in the training group and the validation group.

Variables	Total	Training	Testing	p value
N	11760	8232	3528	—
Gender				0.882
Male	2939	2061	878	
Female	8821	6171	2650	
Age				0.821
<35yr	2406	1677	729	
35-44yr	3727	2611	1116	
45-54yr	3602	2510	1092	
≥55yr	2025	1434	591	
MTD				0.208
<0.75cm	6369	4490	1879	
≥0.75cm	5391	3742	1649	
MF				0.085
Yes	9993	6964	3029	
No	1767	1268	499	
CI				0.427
Yes	6136	4275	1861	
No	5624	3957	1667	
LVI				0.762
Yes	103	74	29	
No	11657	8158	3499	
HT				0.892
Yes	3502	2455	1047	
No	8258	5777	2481	
CLNM				0.536
Yes	5234	3648	1586	
No	6526	4584	1942	

(MTD accounts for maximum tumor diameter. MF, CI, LVI, HT account for multifocality, capsule invasion, lymph-vascular invasion, and Hashimoto’s thyroiditis, respectively. CLNM accounts for central compartment lymph node metastasis).

### 3.6 Prediction nomogram for CLNM in T1-stage PTC

**Figure 6:**
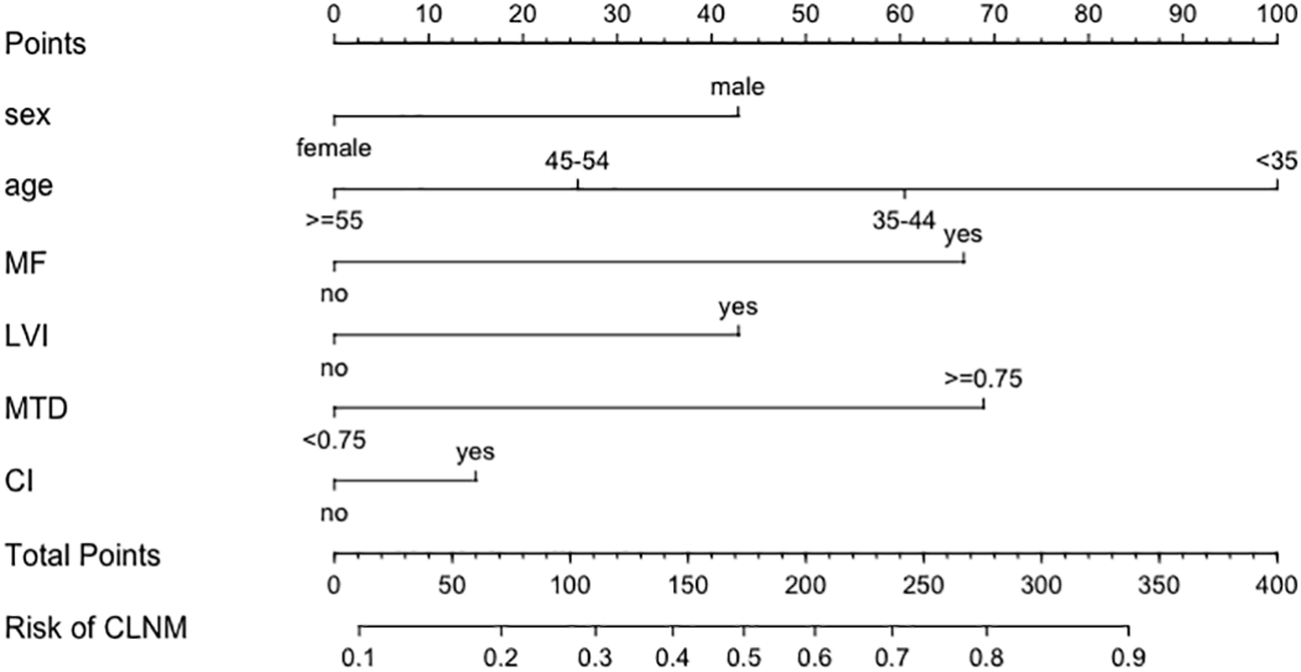
Nomogram of central compartment lymph node metastasis prediction model in patients with T1-stage papillary thyroid carcinoma. (MF, LVI, MTD and CI account for multifocality, lymph-vascular invasion, maximum tumor diameter, and capsule invasion, respectively. How to use the nomogram: To assist readers in applying this tool, the nomogram (**Figure 6**) translates each clinical variable into a point score on the top ‘Points’ scale. For example, a male patient (approx. 43 points) aged 40 (approx. 80 points) with a tumor size of 0.8 cm (approx. 70 points), presenting with multifocality (approx. 67 points), capsule invasion (approx. 15 points), but no lymphovascular invasion (0 points), would accumulate a ‘Total Points’ score of 275. By drawing a vertical line down from 275 on the ‘Total Points’ axis to the ‘Risk of CLNM’ axis, the estimated probability of central lymph node metastasis for this specific patient would be approximately 80%.).

**Figure 7:**
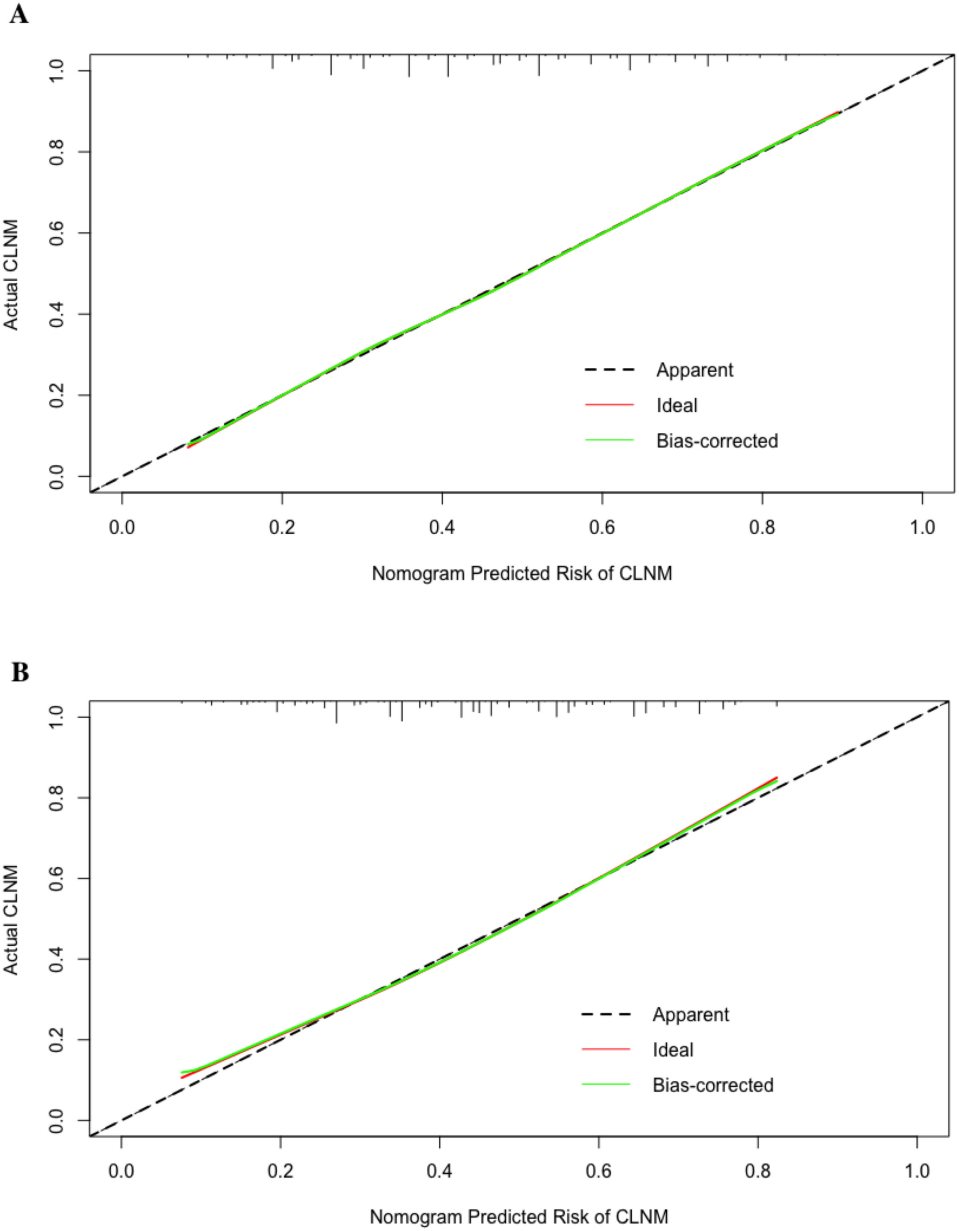
Calibration curve of the nomogram in the training group **(A)** and testing group **(B)**. (CLNM accounts for central compartment lymph node metastasis.

A total of 11,760 patients were divided into training (n=8,232) and testing (n=3,528) group at a 7:3 ratio. The variables distribution between groups showed no significant differences (**Table 3**). A nomogram predicting CLNM risk was constructed using the six identified variables (**Figure 6**). The calibration curve for the training group (**Figure 7A**) showed satisfactory consistency between predicted and actual risk with a C-index of 0.715 (95% CI 0.704-0.726). Validation in the testing group (**Figure 7B**) also demonstrated consistency with a C-index of 0.705 (95% CI 0.688-0.722).

## 4 Discussion

In this study, we provide the first comprehensive description of the clinicopathological landscape of thyroid cancer and its management trends over a 20-year period at a major national cancer center. PTC accounted for 99.3% of all TC cases. Among PTC patients, the male-to-female ratio was about 1:2.8, with an average age of 43.9 years and mean MTD was 1.1cm. The prevalence of MF, CI, LVI and HT was 85.2%, 54.1%, 1.3%, and 27.1%, respectively. Conventional PTC subtype accounted for the vast majority, followed by the follicular subtype, while the remaining subtypes were all less than 1%. CLNM and LLNM rates were 46.4% and 20.9%, respectively. In the past 20 years, both surgical volume and the proportion of TC have increased gradually. The proportion of newly treated PTC has also increased, while the proportion of PTMC has decreased slightly, but still more than 56%. Notably, the rate of CLND increased up to 97%, while the CLNM positivity rate in patients underwent CLND decreased to 55%. For T1-stage PTC, we then identified were male gender, younger age, MTD≥0.75cm, MF, CI, and LVI as risk factors for CLNM. A nomogram was constructed to predict the risk of CLNM in T1-stage PTC. The C-index of the prediction model was 0.715, and the internal validation showed a satisfactory efficacy, with a C-index of 0.705.

This represents the largest longitudinal clinicopathological study of TC to date. The incidence of CLNM in PTC ranges from 20% to 50% (2), and a meta-analysis of 18,741 patients found and LLNM incidence of 20.9% (10), consistent with our finding (46.4% and 20.9%). Compared to a similar 10-year single-institution study by Sui et al. (5), our data showed similar age and HT distributions but significant differences in MTD, MF, CI, and CLNM rates. Regarding trends in diagnosis and treatment modes, both studies noted a rapid increase in CLND rates. However, unlike Sui et al., we observed a slight downward trend in LLND rates and PTMC proportions. This likely reflects the implementation of the Chinese Thyroid Cancer Expert Consensus and ATA Guidelines, which have promoted more standardized diagnosis and treatment. In addition, the widespread use of ultrasound screening, together with the increasing application of fine-needle aspiration (FNA), has improved preoperative risk stratification and patient selection for surgery. Meanwhile, as the National Cancer Center of China, our institution increasingly received patients with malignant or highly suspicious thyroid lesions referred from across the country. It is crucial to acknowledge the inherent selection bias in our cohort. Therefore, the marked rise in the proportion of thyroid cancer cases among surgically treated patients likely reflects the combined effects of evolving guideline-based practice, improved preoperative diagnostic triage, and referral concentration in a cancer-specialized center, rather than a simple epidemiological shift in the general hospital setting.

It should be acknowledged that a substantial proportion of patients in our cohort underwent central neck dissection, including prophylactic procedures, which differs from routine practice in many Western countries. This reflects historical and regional surgical preferences in East Asia, where prophylactic central lymph node dissection has been more commonly performed. Therefore, the temporal decrease in the proportion of pathologically confirmed CLNM among patients undergoing CLND should be interpreted with caution, as it likely reflects the expanding use of prophylactic CLND and the inclusion of more clinically node-negative patients in the dissected cohort, rather than a true decline in the biological incidence of CLNM. Importantly, this practice provides a unique real-world opportunity to comprehensively evaluate the true incidence and predictors of occult central lymph node metastasis, which are often underestimated in cohorts where central lymph nodes are not routinely assessed. Therefore, while differences in surgical strategy may limit direct extrapolation, our findings offer complementary evidence that may enhance understanding of central nodal disease biology in papillary thyroid carcinoma.

Previous studies have identified male, younger age, larger MTD, CI, MF, LVI, bilateral tumors, calcifications on ultrasound as risk factors for CLNM (11–14). Our findings in T1-stage PTC patients align with these reports. While Xu et al. (9) suggested HT as a protective factor, our study found no significant correlation between HT and CLNM in this specific cohort.

To our knowledge, this is the largest retrospective study developing a CLNM prediction nomogram for PTC. Previous studies have reported the prediction model of CLNM in PTC, especially PTMC patients. Tian et al. (15) used US signature, US-reported LN status to establish a nomogram for CLNM risk prediction, but the performance of this model in elder women was not as good as in men and younger women. However, the ultrasound results are operator-dependent, which might result in a significant bias. Gao et al., (16) established a CLNM prediction model based on the clinical data of PTC patients. Although the C-index was 0.715, only three variables, MTD, microcalcification, and Thyroid Globin Anti-body (TGAb), were finally included in the nomogram with a small sample size (n=296). Our study included 11,760 patients, ensuring robust statistical power. The resulting nomogram achieved satisfactory prediction performance (C-index > 0.7).

Significantly, we identified an optimal MTD cutoff value of 0.75 cm for predicting CLNM. Although the 2016 Chinese expert consensus lists MTD ≥ 0.6 cm as a relative indication for surgery (17), current international guidelines generally accept tumors ≤ 1.0 cm (PTMC) as candidates for AS due to favorable outcomes (2, 18–20). However, our findings suggest that tumor size > 0.75 cm is an independent risk factor for CLNM. This finding underscores the biological heterogeneity within the PTMC category itself. While the 1.0 cm threshold is traditionally used to define PTMC and often serves as a universal criterion for Active Surveillance, our statistically derived 0.75 cm cutoff reveals a potential high-risk clinical ‘gray zone’ (0.75 cm to 1.0 cm). Tumors in this specific range, although still classified as microcarcinomas, may exhibit more aggressive behavior and a higher propensity for early lymphatic spread than their smaller counterparts (<0.75 cm). This raises a critical consideration for the current AS criteria: the “one-size-fits-all” 1.0 cm threshold may overlook the elevated metastatic risk in the 0.75–1.0 cm subgroup. Consequently, we suggest that AS decisions for patients with tumors > 0.75 cm should be made with greater caution. Although they qualify as PTMC, these patients might benefit from more rigorous preoperative assessment or a lower threshold for surgical intervention compared to those with smaller tumors.

Regarding age, we stratified patients into four groups (19-35, 35-44, 45-54, and ≥ 55 years) to refine the model. We observed that CLNM risk decreased significantly with increasing age, consistent with Yasuhiro Ito et al. (18). This implies that younger patients, particularly those with tumors > 0.75 cm, represent a higher-risk cohort. Our nomogram integrates these critical variables, offering a practical tool to accurately screen patients.

We acknowledge that several variables included in the nomogram, such as lymphovascular invasion and capsule invasion, are pathological features available only after surgery. Therefore, we explicitly emphasize that the proposed nomogram is not intended to guide the initial extent of surgical management or the decision to perform prophylactic central neck dissection. This limitation is inherent to the model’s design; several of its strongest predictors, such as LVI and CI, require comprehensive histopathological examination of the resected tumor and are currently unattainable with high precision in the preoperative phase. Consequently, this tool is strictly defined as a postoperative risk-augmentation instrument. Instead, this model must strictly serve as a postoperative risk stratification tool that complements conventional pathological staging systems. It may help identify patients with T1-stage papillary thyroid carcinoma who are at higher risk of central lymph node metastasis and who may benefit from closer surveillance, tailored follow-up strategies, or consideration of adjuvant therapy. Furthermore, while current clinical guidelines, such as the ATA recommendations, provide robust frameworks for overall disease management based on broad clinicopathological risk groups, our nomogram complements these guidelines by offering a more granular, individualized, and quantitative risk assessment. By integrating specific microscopic features (e.g., lymphovascular and capsule invasion) into a unified scoring system, this tool can assist clinicians in refining postoperative risk stratification for patients who might otherwise fall into indeterminate risk categories.

Rather than emphasizing the novelty of individual predictors, our nomogram enables individualized risk estimation through combined modeling, which may provide greater clinical utility than isolated risk factor assessment. Although the risk factors identified in this study, including age, sex, tumor size, multifocality, lymphovascular invasion, and capsule invasion, have been reported previously, the clinical value of our work lies in the integration and quantification of these variables within a large, contemporary cohort spanning two decades.

Some limitations exist in our study. Firstly, this is a retrospective study, and the result should be further validated in prospective studies. Secondly, as a single institutional study, we only performed the internal testing. External validation should be performed to further test the efficacy of the predictive nomogram. Thirdly, as a single-center study at a national referral center, selection bias regarding case complexity is unavoidable. Finally, we lack data on preoperative ultrasound (US) features and fine-needle aspiration (FNA), especially the BRAF mutation status, which are known to be associated with CLNM (21). We aim to integrate these in future research.

## 5 Conclusions

In conclusion, this study described the evolving clinicopathological characteristics and management trends of thyroid disease over a 20-year period at a national cancer center. Male sex, younger age, tumor size ≥0.75 cm, multifocality, capsule invasion, and lymphovascular invasion were identified as independent risk factors for central lymph node metastasis in T1-stage papillary thyroid carcinoma. The proposed nomogram may serve as a pathology-based postoperative risk stratification tool to assist individualized patient management and follow-up.

## Data Availability

The original contributions presented in the study are included in the article/**Supplementary Material**. Further inquiries can be directed to the corresponding author.

## Supplementary material

Supplementary Table 1 Basic information of the population by year.

Supplementary Figure 1 Pathological subtype of papillary thyroid carcinoma.

Supplementary Figure 2 Receiver operating characteristic curve (ROC) between central compartment lymph node metastasis (CLNM) and maximum tumor diameter (MTD).
