# Supplementary figures and images for: Clinicopathological landscape and management trends of thyroid carcinoma over two decades: a single-institution study and risk stratification of central lymph node metastasis in T1 papillary thyroid carcinoma
Source: Front Endocrinol (Lausanne). 2026 Apr 30;17:1776293. doi: 10.3389/fendo.2026.1776293 (PMC13171347; doi:10.3389/fendo.2026.1776293)

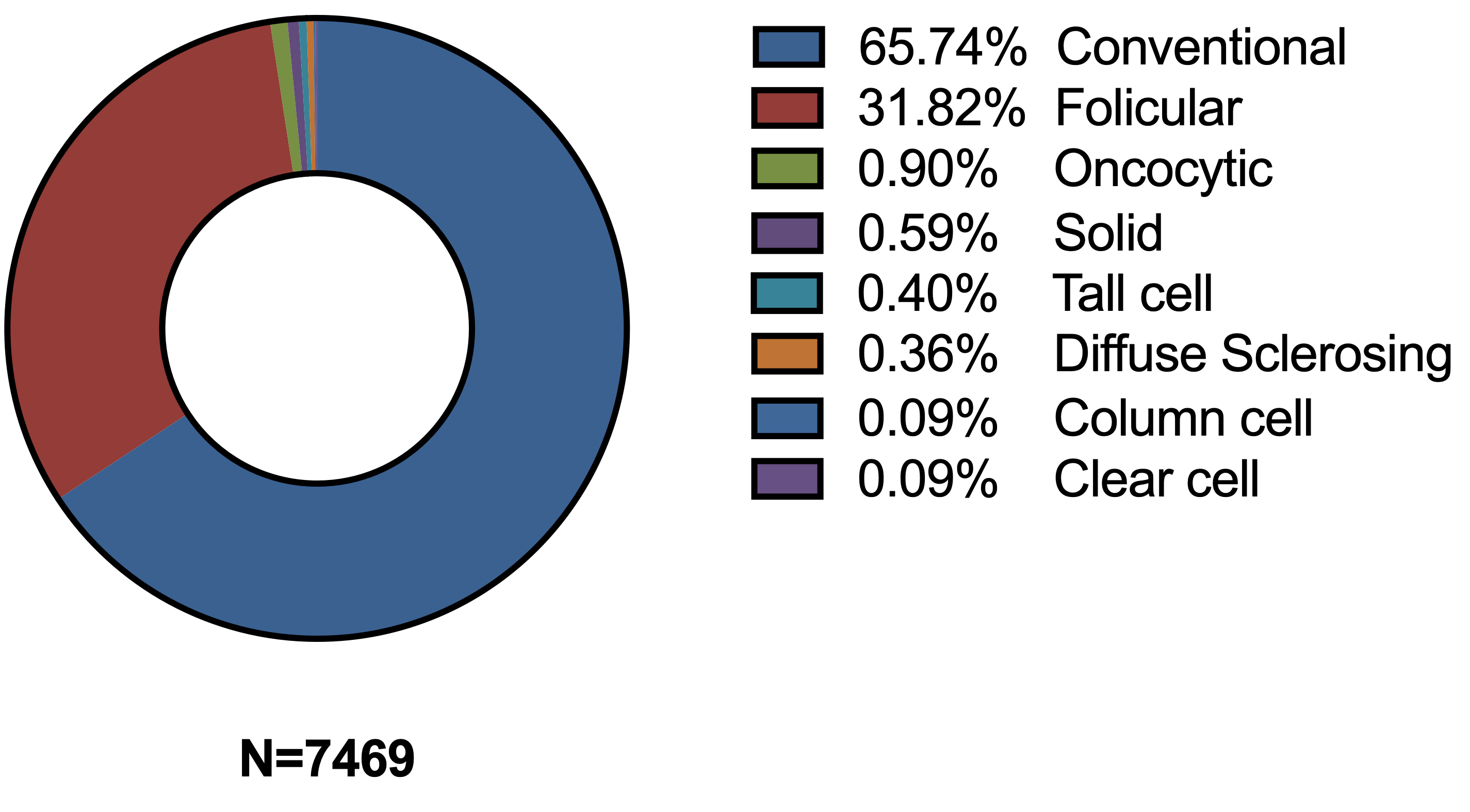

Supplement: Supplementary Table 1 — Basic information of the population by year. [file Image1.tiff]

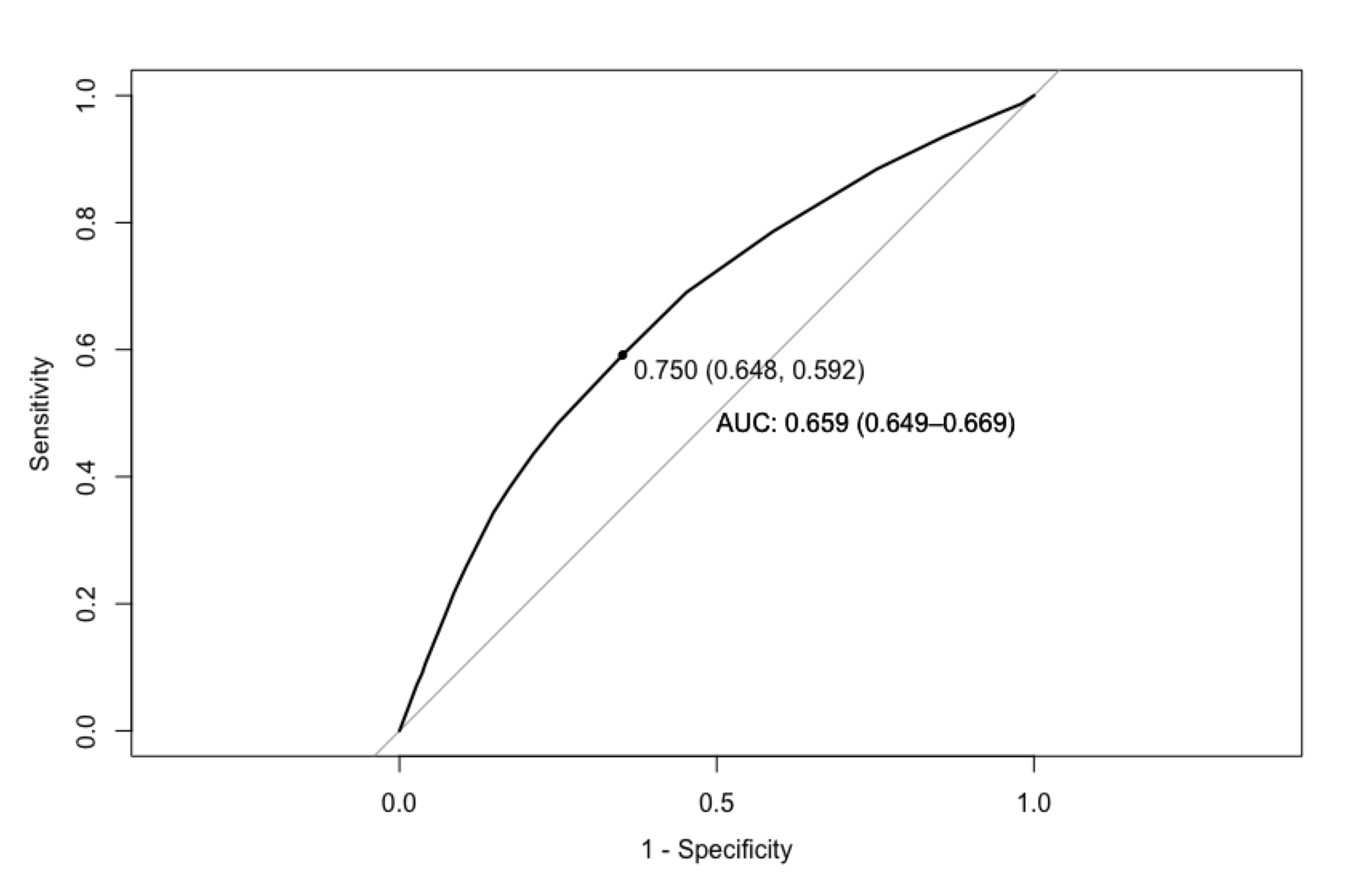

Supplement: Supplementary Figure 1 — Pathological subtype of papillary thyroid carcinoma. [file Image2.tiff]
