# Supplementary material for: Clinicopathological landscape and management trends of thyroid carcinoma over two decades: a single-institution study and risk stratification of central lymph node metastasis in T1 papillary thyroid carcinoma
Source: Front Endocrinol (Lausanne). 2026 Apr 30;17:1776293. doi: 10.3389/fendo.2026.1776293 (PMC13171347; doi:10.3389/fendo.2026.1776293)
Supplement: Supplementary Figure 2 — Receiver operating characteristic curve (ROC) between central compartment lymph node metastasis (CLNM) and maximum tumor diameter (MTD). [file Table1.docx]

Supplemental Table 1 Basic information of the population by year.

| Year | | Total | 1999 | 2000 | 2001 | 2002 | 2003 | | 2004 | | 2005 | | 2006 | | 2007 | | 2008 | | 2009 | | 2010 | | 2011 | | 2012 | | 2013 | | 2014 | | 2015 | | 2016 | | 2017 | | 2018 | |
| --- | --- | --- | --- | --- | --- | --- | --- | --- | --- | --- | --- | --- | --- | --- | --- | --- | --- | --- | --- | --- | --- | --- | --- | --- | --- | --- | --- | --- | --- | --- | --- | --- | --- | --- | --- | --- | --- | --- |
| N | Total | 34420 | 329 | 450 | 586 | 529 | 484 | | 662 | | 927 | | 1013 | | 1156 | | 1285 | | 1473 | | 1690 | | 1847 | | 2092 | | 2746 | | 3457 | | 3224 | | 3612 | | 3084 | | 3774 | |
|  | Malignance | 22919 | 129 | 155 | 196 | 113 | 97 | | 144 | | 226 | | 260 | | 325 | | 456 | | 576 | | 834 | | 1117 | | 1452 | | 2061 | | 2515 | | 2623 | | 2862 | | 3030 | | 3748 | |
|  | First malignance | 18917 | 129 | 155 | 196 | 96 | 77 | | 127 | | 204 | | 240 | | 301 | | 421 | | 544 | | 810 | | 1078 | | 1376 | | 1875 | | 1886 | | 1884 | | 1651 | | 2177 | | 3690 | |
| Histological Type | PTC | 18785 | 112 | 139 | 171 | 96 | 77 | | 126 | | 202 | | 238 | | 294 | | 420 | | 540 | | 810 | | 1064 | | 1372 | | 1870 | | 1885 | | 1881 | | 1648 | | 2160 | | 3680 | |
|  | FTC | 18 | 4 | 5 | 8 | 0 | 0 | | 0 | | 0 | | 0 | | 0 | | 0 | | 0 | | 0 | | 0 | | 0 | | 0 | | 0 | | 0 | | 0 | | 1 | | 0 | |
|  | MTC | 37 | 6 | 9 | 9 | 0 | 0 | | 0 | | 0 | | 0 | | 0 | | 0 | | 0 | | 0 | | 3 | | 0 | | 0 | | 0 | | 0 | | 0 | | 8 | | 2 | |
|  | PDC | 24 | 2 | 1 | 5 | 0 | 0 | | 1 | | 1 | | 0 | | 2 | | 1 | | 0 | | 0 | | 3 | | 1 | | 1 | | 0 | | 0 | | 0 | | 2 | | 4 | |
|  | ATC | 28 | 4 | 1 | 2 | 0 | 0 | | 0 | | 1 | | 1 | | 3 | | 0 | | 1 | | 0 | | 5 | | 0 | | 0 | | 1 | | 1 | | 1 | | 4 | | 3 | |
|  | PTC+MTC | 2 | 1 | 0 | 0 | 0 | 0 | | 0 | | 0 | | 0 | | 0 | | 0 | | 0 | | 0 | | 0 | | 0 | | 0 | | 0 | | 0 | | 0 | | 1 | | 0 | |
|  | PTC+FTC | 23 | 0 | 0 | 1 | 0 | 0 | | 0 | | 0 | | 1 | | 2 | | 0 | | 3 | | 0 | | 3 | | 3 | | 4 | | 0 | | 2 | | 2 | | 1 | | 1 | |
| PTMC | | N | 16510 | 107 | 137 | 167 | 112 | 91 | 140 | | 215 | | 249 | | 304 | | 423 | | 521 | | 814 | | 997 | | 1291 | | 1609 | | 1811 | | 1731 | | 1808 | | 1792 | | 2191 | |
| Gender | Male | 4908 | 31 | 38 | 39 | 20 | 15 | | 30 | | 51 | | 66 | | 79 | | 125 | | 163 | | 190 | | 310 | | 355 | | 492 | | 452 | | 500 | | 433 | | 559 | | 960 | |
|  | Female | 13877 | 81 | 101 | 132 | 76 | 62 | | 96 | | 151 | | 172 | | 215 | | 295 | | 377 | | 620 | | 754 | | 1017 | | 1378 | | 1433 | | 1381 | | 1215 | | 1601 | | 2720 | |
| Age/years old | Mean | 43.9 | 40.2 | 43.5 | 47.8 | 47.4 | 51.6 | | 40.6 | | 45.5 | | 46.1 | | 47.9 | | 46.0 | | 45.6 | | 45.7 | | 44.3 | | 44.5 | | 43.9 | | 43.3 | | 43.6 | | 43.5 | | 43.1 | | 44.3 | |
|  | SD | 11.1 | 12.9 | 9.4 | 14.5 | 12.7 | 12.2 | | 6.9 | | 15.8 | | 11.9 | | 12.1 | | 11.3 | | 12.0 | | 11.5 | | 10.8 | | 10.4 | | 10.8 | | 10.5 | | 10.8 | | 11.4 | | 11.2 | | 11.4 | |
| MTD/cm | Mean | 1.1 | 0.9 | 0.9 | 1.6 | 0.5 | 1.7 | | 0.7 | | 1.3 | | 0.9 | | 0.8 | | 0.9 | | 0.8 | | 0.6 | | 1.0 | | 0.9 | | 1.0 | | 1.1 | | 1.1 | | 1.2 | | 1.2 | | 1.3 | |
|  | SD | 1.2 | 1.0 | 1.4 | 2.1 | 0.3 | 2.3 | | 0.5 | | 1.7 | | 1.5 | | 1.1 | | 1.2 | | 0.8 | | 0.7 | | 1.1 | | 0.9 | | 1.0 | | 1.0 | | 1.1 | | 1.1 | | 1.3 | | 1.4 | |
| Lymph Node | CLNM | 8710 | 38 | 57 | 59 | 27 | 24 | | 49 | | 64 | | 76 | | 85 | | 155 | | 206 | | 322 | | 418 | | 598 | | 812 | | 847 | | 926 | | 849 | | 1110 | | 1988 | |
|  | CLND | 15661 | 55 | 76 | 77 | 33 | 36 | | 64 | | 91 | | 116 | | 133 | | 225 | | 322 | | 544 | | 740 | | 1070 | | 1561 | | 1637 | | 1723 | | 1576 | | 2014 | | 3568 | |
|  | LLNM | 3925 | 47 | 67 | 77 | 44 | 21 | | 50 | | 60 | | 75 | | 81 | | 113 | | 136 | | 247 | | 225 | | 283 | | 320 | | 281 | | 297 | | 322 | | 447 | | 732 | |
|  | LLND | 5666 | 58 | 81 | 85 | 46 | 24 | | 55 | | 80 | | 93 | | 106 | | 146 | | 183 | | 359 | | 363 | | 426 | | 501 | | 430 | | 448 | | 461 | | 663 | | 1058 | |
| CI | | 10172 | 21 | 37 | 58 | 15 | 23 | | 59 | | 73 | | 108 | | 149 | | 206 | | 292 | | 418 | | 630 | | 835 | | 1111 | | 1061 | | 1102 | | 940 | | 1099 | | 1935 | |
| MF | | 16001 | 92 | 118 | 145 | 76 | 53 | | 104 | | 173 | | 197 | | 236 | | 343 | | 480 | | 695 | | 880 | | 1142 | | 1553 | | 1539 | | 1594 | | 1437 | | 1849 | | 3295 | |
| LVI | | 244 | 0 | 2 | 3 | 2 | 1 | | 3 | | 4 | | 2 | | 3 | | 1 | | 6 | | 5 | | 8 | | 14 | | 14 | | 11 | | 22 | | 35 | | 40 | | 68 | |
| HT | | 5091 | 12 | 10 | 15 | 7 | 7 | | 14 | | 28 | | 33 | | 31 | | 63 | | 111 | | 169 | | 251 | | 374 | | 467 | | 566 | | 594 | | 554 | | 666 | | 1119 | |

*PTC, FTC, MTC, PDC, and ATC account for papillary thyroid carcinoma, follicular thyroid carcinoma, medullary thyroid carcinoma, poorly differentiated thyroid carcinoma, and anaplastic thyroid carcinoma, respectively. SD accounts for standard deviation. MTD account for* *maximum tumor diameter. CLNM, CLND, LLNM, and LLND account for central compartment lymph node metastasis, central compartment* *lymph node dissection, lateral compartment* *lymph node metastasis, and lateral compartment lymph node dissection, respectively.*
